# Microcirculatory effect of hyperbaric oxygen therapy in septic patients

**DOI:** 10.1186/cc9704

**Published:** 2011-03-11

**Authors:** F Ferré, S Silva, J Ruiz, A Mari, O Mathe, P Sanchez-Verlaan, B Riu-Poulenc, O Fourcade, M Génestal

**Affiliations:** 1 University Teaching Hospital Purpan, Toulouse, France

## Introduction

Reduced microvascular perfusion has been implicated in organ dysfunction and multiple organ failure associated with severe sepsis. Near-infrared spectroscopy (NIRS) can provide a non-invasive estimation of local tissue oxygenation (StO_2_) related to microvascular circulation. Previous investigators have reported a prognosis value of StO_2 _measurements realized during severe sepsis. Hyperbaric oxygen (HBO) is recommended as an associated treatment during soft-tissue severe infection. Interestingly, a microcirculation improvement has been reportedly identified in septic animals treated by HBO. The aim of this study is to evaluate the microcirculatory effect of HBO therapy in septic patients assessing dynamics changes in StO_2_.

## Methods

**Figure 1:**
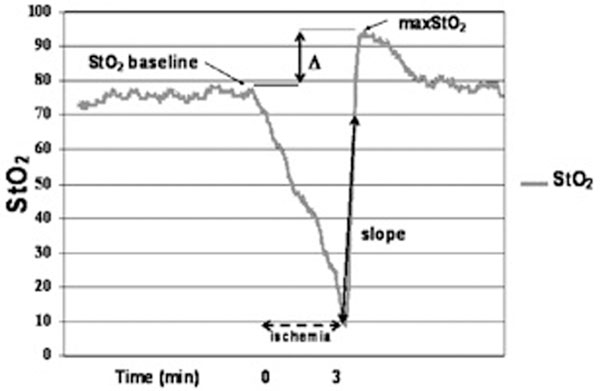
**Dynamic changes of StO_2 _during ischemic challenge**.

A prospective study over 1 year investigating 14 septic shock patients secondary to a soft-tissue infection. A concomitant microcirculation (for example, dynamic changes in StO_2_), macro-circulation and metabolic assessment was performed before and after each HBO session (for the first three). Thenar eminence StO_2 _was measured continuously by NIRS during a vascular occlusion test: a 3-minute transient ischemia inflating an arm cuff 50 mmHg above the systolic arterial pressure (Figure 1). Primary end point: StO_2 _reperfusion slope variation induced by HBO.

## Results

The reperfusion slopes on day 1 were lower in nonsurvivors compared with survivors (*P *= 0.05). HBO increases cardiac output (*P *= 0.003) and reduces arterial blood lactate (*P *= 0.001). HBO improves post-ischemic microcirculatory parameters: hyperemic area (*P *= 0.01), ΔStO_2 _(*P *= 0.02), maximum StO_2 _(*P *= 0.04) and tends to improve reperfusion slope (*P *= 0.1). A significant negative correlation between reperfusion slope and blood lactate was observed. No correlation between macrohemodynamic and microcirculatory parameters, including baseline StO_2 _with ScvO_2_, was observed.

## Conclusions

If microvascular dysfunction is the key to the development of multiple organ failure in sepsis, the microcirculation should be a key therapeutic target. Our data confirm a good predictive value for outcome of the StO_2 _reperfusion slope at admission. Originally, we demonstrated a post-ischemic NIRS parameter improvement by HBO therapy. This microcirculatory effect, independent of the HBO action on systemic hemodynamic parameters, was associated with a significant reduction of arterial lactate, a major prognostic factor in septic patients. These variations are probably due to capillaries recruitment induced by microvascular reactivity modifications.
